# Direct comparison of the four aldehyde oxidase enzymes present in mouse gives insight into their substrate specificities

**DOI:** 10.1371/journal.pone.0191819

**Published:** 2018-01-25

**Authors:** Gökhan Kücükgöze, Silke Leimkühler

**Affiliations:** Department of Molecular Enzymology, Institute of Biochemistry and Biology, University of Potsdam, Potsdam, Germany; Universidade Nova de Lisboa, PORTUGAL

## Abstract

Mammalian aldehyde oxidases (AOXs) are molybdo-flavoenzymes which are present in many tissues in various mammalian species, including humans and rodents. Different species contain a different number of AOX isoforms. In particular, the reasons why mammals other than humans express a multiplicity of tissue-specific AOX enzymes is unknown. In mouse, the isoforms mAOX1, mAOX3, mAOX4 and mAOX2 are present. We previously established a codon-optimized heterologous expression systems for the mAOX1-4 isoforms in *Escherichia coli* that gives yield to sufficient amounts of active protein for kinetic characterizations and sets the basis in this study for site-directed mutagenesis and structure-function studies. A direct and simultaneous comparison of the enzymatic properties and characteristics of the four enzymes on a larger number of substrates has never been performed. Here, thirty different structurally related aromatic, aliphatic and N-heterocyclic compounds were used as substrates, and the kinetic parameters of all four mAOX enzymes were directly compared. The results show that especially mAOX4 displays a higher substrate selectivity, while no major differences between mAOX1, mAOX2 and mAOX3 were identified. Generally, mAOX1 was the enzyme with the highest catalytic turnover for most substrates. To understand the factors that contribute to the substrate specificity of mAOX4, site-directed mutagenesis was applied to substitute amino acids in the substrate-binding funnel by the ones present in mAOX1, mAOX3, and mAOX2. An increase in activity was obtained by the amino acid exchange M1088V in the active site identified to be specific for mAOX4, to the amino acid identified in mAOX3.

## Introduction

Aldehyde oxidases (AOX; EC 1.2.3.1) are molybdo-flavoenzymes present in the cytosol of various tissues and animal species [1]. AOXs are widely distributed from insects to humans and the numbers of AOX present in one organism is variable. Humans and chimpanzees are endowed with one active enzyme (AOX1), while rodents are characterized by the largest number of four AOX isoenzymes. Mice are expressing the AOX isoenzymes from a gene clustering on chromosome 1 in the order of *Aox1*, *Aox3*, *Aox4*, *Aox2* [2]. The isoenzymes of AOX were evolved by gene duplication and suppression events from a common ancestral XOR gene and the amino acid similarity among them is approx. 60% [3, 4].

The four mouse AOX isoenzymes are expressed in a tissue-specific manner, indicating that each isoenzyme may take a special role in recognizing distinct substrates in different tissues [4]. Isoform mAOX1 is mainly expressed in liver, lung, and testis and its tissue distribution is superimposable to that of mAOX3 [5]. Isoform mAOX4 is present in the skin, epithelial lining of the oral cavity and the esophagus. The major source for mAOX4 is the Harderian gland, an organelle located behind the eye bulb in many vertebrates, which is not present in humans. It is an important organelle, which is involved in thermoregulation and lubrication of the eye surface. Considering the fact that no other AOX isoenzyme is present in the Harderian gland, distinct compounds might be recognized by mAOX4 as substrate to contribute to the role of the Harderian gland in the vertebral body [5]. Indeed, deletion of mAOX4 in the Harderian gland caused perturbations in the circadian rhythm, resulting in reduced locomotor activity, resistance to diet-induced obesity and hepatic steatosis in mice [6]. In a recent study, tryptophan and 5-hydroxy-indole-acetic acid were postulated to be the endogenous substrate for mAOX4 in the Harderian gland of the mouse [6]. In contrast, expression of mAOX2 is highly restricted to the nasal cavity. High levels of mRNA transcripts and proteins are detectable especially in Bowman’s gland, which is responsible for the production of mucus to moisturize the olfactory mucosa [7].

Overall, very little is known about the physiological substrates of AOX enzymes including human AOX1 or the differences in the AOX isoforms in other species [8]. Recently, various reports combined computational and experimental studies to investigate the prediction of substrates cleared by hAOX1 [9, 10]. AOXs in general are endowed with the ability to oxidize a broad range of substrates among which are not only aldehydes (e.g. vanillin and benzaldehyde) but also the compounds containing aza-heterocycles (e.g. phthalazine and N^1^-methylnicotinamide) [4]. In fact, the ability to oxidize N-heterocycles is a role of AOX with emerging importance for the metabolism and drugs in humans [11]. For instance, hAOX1 takes a potent role in the clearance of drugs such as zaleplon, methotrexate and ziprasidone [12–14]. Additionally, hAOX may have an overlooked role in the oxidation of bulky lipophilic compounds such as tamoxifen, due to its ability to oxidize aldehydes, which are intermediate compounds during the oxidation of alcohols to carboxylic acids [15].

In mice, the mAOX1, mAOX3, mAOX4 and mAOX2 proteins are highly similar to each other in terms of domain organization and cofactor insertion as indicated by around 60% amino acid sequence similarity among them [6]. They are 300 kDa homodimeric enzymes and each monomeric subunit is characterized by three subdomains: an N-terminal 2x[2Fe2S] cluster containing domain, a central FAD-containing domain and a C-terminal molybdenum cofactor (Moco) containing and substrate binding domain [1]. After substrate conversion, the electrons are transferred to FAD via the two FeS centers as a result of intramolecular electron transfer. Oxygen is the final electron acceptor for AOX. A sulfido-ligand at the molybdenum atom has been revealed to be essential for the activity of the enzymes [16].

Crystal structures for hAOX1 and mAOX3 are available. The first crystal structure of a mammalian aldehyde oxidase was determined for the mAOX3 enzyme (PDB ID: 3ZYV) to 2.9Å [17, 18]. More recently the crystal structure of the human enzyme hAOX1, in substrate free (PDB ID: 4UHW) and in complex with the substrate phthalazine and the inhibitor thioridazine (PDB ID: 4UHX) was additionally solved to 2.6 Å and 2.7 Å resolution, respectively [19]. In both cases, crystals were prepared using the recombinant protein expressed in *E*. *coli*. The mAOX3 structure was insufficient to study the hAOX1 metabolism due to the low sequence identity (60%) between the two proteins and the presence of several different isoforms in mouse and other animals, as previously mentioned. The mouse and human AOX crystal structures possess nevertheless a high overall similarity but have marked differences at the FAD site in addition to the Mo active site and substrate funnel which account for some of the different species specificities observed [19]. The structures of the mouse isoforms mAOX1, mAOX4 and mAOX2 are not available so far.

In the absence of the crystal structures for the other mouse isoforms (AOX1, AOX2 and AOX4) the crystal structure of mAOX3 was previously used as a model in a computational study for evaluating the factors that modulate substrate specificity and activity in the different isoenzymes [20]. Major differences between the several isoforms were predicted from the modeling at the protein surface and in the substrate-binding site region. The results from the computational studies suggested that the mAOX1 isoform has the wider specificity region being able to accept a wider range of substrates with variable shape, size and nature while, in contrast, the mAOX4 isoform, with the narrowest specificity region, would bind only smaller and more hydrophobic substrates. It has been predicted that the substrate specificities of mAOX2 and mAOX4 overlap while those of mAOX3 and mAOX1 are also similar to each other. Direct comparison of the substrate specificities of the purified enzymes have not been performed so far. By determining the substrate specificities of each mouse isoform the question could be addressed why rodents contain different tissue-specific, while other species like humans contain only one isoform. This question is of relevance for the use of mouse as an animal model in the context of drug discovery programs. Previous reports, however, showed that mice and other rodents are unlikely to represent suitable models for drug metabolism predictions in humans based on differences in the isoforms and differences to the human enzyme in substrate specificity and superoxide production [21].

Recently, we established and optimized an efficient *s*ystem for the heterologous expression of all four mouse AOX enzymes in *Escherichia coli* [21]. Using this system we are now able to directly compare the activities of all four mAOX enzymes using different substrates. So far, the initial characterizations showed that the enzymes are not only different at their active sites but also at the FAD site, as indicated by the differences in the superoxide production levels and the interaction with NADH [21]. In this study, 30 different structurally related aromatic and aliphatic compounds were used as substrates, and the kinetic parameters of all four mAOX enzymes were directly compared for the first time. The results show that especially mAOX4 displays a different substrate selectivity as compared to the other three mAOX enzymes. To understand the factors that contribute to the substrate specificity of mAOX4, we have additionally substituted the amino acids in the substrate-binding funnel of this enzyme to the ones present in mAOX1, mAOX3, and mAOX2 by site-directed mutagenesis. Especially one amino acid exchange in the substrate-binding site resulted in an increased activity of mAOX4.

## Results and discussion

### Steady-state kinetics of mAOX isoenzymes with different substrates

In previous studies, the mAOX isoenzymes have been characterized separately after purification from their native tissues using mouse livers, the Harderian gland or the Bowmans’s gland [5, 22–25]. In these studies the risk remained of cross-contaminations with the other isoenzymes. Further, the yield was usually low and several purification steps were necessary to obtain a highly pure enzyme. The recently established expression system for each mAOX isoform in *E*. *coli* now gives rise to a high yield of enzymes in a reproducible manner and without the cross-contamination of the other AOX isoenzymes enabling direct comparison studies, which were not possible before [21].

To directly compare the substrate specificity and kinetic properties of all mAOX isoforms using several different substrates, mAOX1, mAOX3, mAOX4 and mAOX2 (in the order of their arrangement on the chromosome [6]) were characterized in detail after purification. Kinetic parameters of mAOX1, mAOX3, mAOX4, and mAOX2 were determined for each substrate with the goal to identify the substrate specificity of each enzyme and to compare differences based on structural differences at the molybdenum active site and the substrate-binding funnel. Kinetic parameters were determined by steady-state kinetics using DCPIP as an electron acceptor and varying concentrations of 30 different aromatic and aliphatic substrates in addition to selected N-heterocyclic compounds. The substrates used in this study are divided into azo-heterocyclic compounds or substrates containing an aldehyde group (benzaldehydes, alkyl aldehydes and structurally related cinnamaldehydes). The *K*_*M*_ and *k*_*cat*_ values were determined by non-linear regression based on the rate of DCPIP reduction at 600 nm. The molybdenum saturation of each AOX enzyme was determined as reported previously [26] and was used to normalize the kinetic constants for a 100% molybdenum saturation level for a better comparability of the kinetic parameters for the four AOX enzymes. Analysis of the substrate specificity of mouse AOX enzymes showed that enzymes can convert of a wide spectrum of substrates and that indeed differences among the substrate specificities of the four mAOX enzymes exist, however, with differences as predicted by computational studies previously. The kinetic data for each group of substrates are presented below and discussed separately.

### Aromatic aldehydes containing a benzyl-group

**Fig 1:**
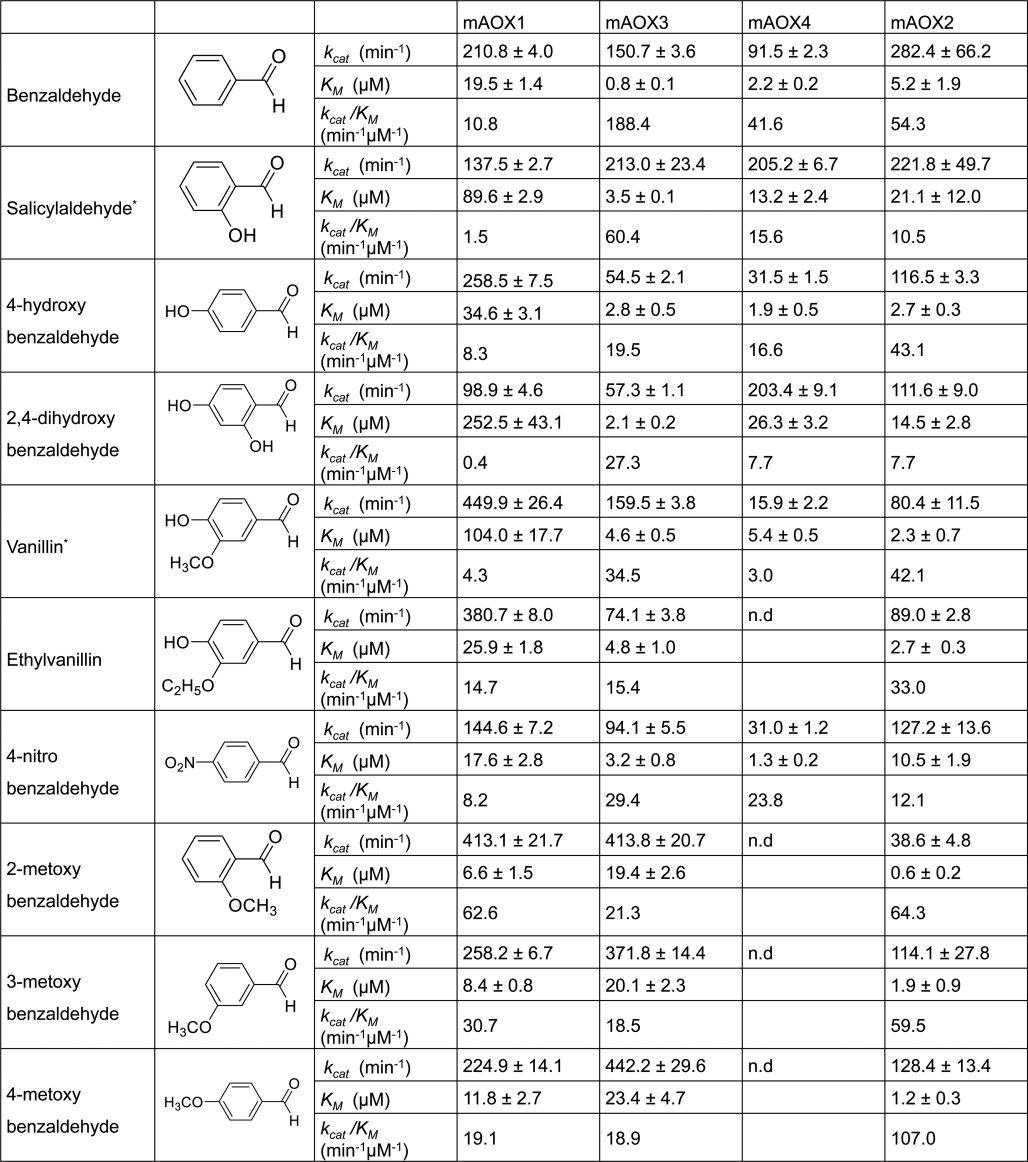
Steady-state kinetic parameters for mAOX1-4 with aromatic aldehydes as substrates containing a benzyl-group. Apparent steady-state kinetic parameters were recorded in 50 mM Tris-HCl, 200 mM NaCl, and 1 mM EDTA (pH 8.0) in the presence of 100 μM DCPIP as electron acceptor. The substrate concentrations were varied around 0.5 and 10 times the K_M_. The chemical structure of each substrate is shown in the Fig. The values were corrected to a molybdenum saturation of 100% for each mAOX variant for a better comparability. Kinetic Data are mean values from three independent measurements (±S.D.). n.d. = no activity detectable.

As first group of substrates for mAOX1-4 we tested aromatic aldehydes containing a benzyl-group with different substituents. The substrates are listed in Fig 1. While all substrates were converted by mAOX1, mAOX3 and mAOX2, only mAOX4 was unable to use all methoxybenzaldehydes and ethylvanillin as substrates. In general, mAOX1 displayed the highest activity with all benzaldehyde derivates, while the *K*_*M*_ was also higher in comparison to the other mAOX isoenzymes. In particular, 2-methoxybenzaldehyde was the best substrate for mAOX1 in terms of catalytic efficiency with a value of 62.6 min^-1^μM^-1^. 3- and 4-methoxybenzaldehydes were also good substrates for mAOX1 with catalytic efficiencies of 30.7 min^-1^μM^-1^ and 19.1 min^-1^μM^-1^, respectively. While vanillin was recognized by mAOX1 with the highest turnover number (449.9 min^-1^), the *K*_*M*_ value was 15 times higher than that for other 2-methoxybenzadehydes, which resulted in a low catalytic efficiency of 4.3 min^-1^μM^-1^ for this aldehyde. For mAOX2, the best substrates were methoxybenzaldehydes, benzaldehyde, 4-hydroxybenzaldehyds, and vanillin in terms of catalytic efficiencies with values around 30 min^-1^μM^-1^ for all the substrates. For mAOX3, the substrate with the highest catalytic efficiency is benzaldehyde (188.4 min^-1^μM^-1^), followed by salicylaldehyde (60.4 min^-1^μM^-1^) and vanillin (34.5 min^-1^μM^-1^). Benzaldehyde was also the most catalytic efficient substrate for mAOX4 (41.6 min^-1^μM^-1^) while the *k*_*cat*_ for salicylaldehyde was almost two times higher than that for benzaldehyde.

The effect of hydroxy-groups on the selectivity by mAOX1-4 was analyzed by comparing 2-hydroxybenzaldehyde (salicylaldehyde), 4-hydroxybenzyldehyde, and 2,4-dihydroxybenzaldehyde. Generally, a high *k*_*cat*_ value for salicylaldehyde of >200 min^-1^ was obtained for mAOX3, mAOX4, and mAOX2. However, when the hydroxyl group was present at position 4 (4-hydroxybenzaldehyde), the turnover number for these enzymes was largely decreased about a factor of 2–6. In contrast, the turnover number of mAOX1 was increased almost two times with 4-hydroxybenzaldehyde in comparison to salicylaldehyde. However, when 2,4-dihydroxybenzaldehyde was used as a substrate, the *K*_*M*_ for mAOX1, mAOX4, and mAOX2 was decreased to the similar values as obtained for salicylaldehyde as substrate. Interestingly, the *K*_*M*_ for 2,4-dihydroxybenzaldehyde of mAOX3 was not affected by the hydroxyl groups at different positions of the substrate, revealing a higher flexibility of the substrate-binding site of this enzyme. The *k*_*cat*_ values of mAOX4 with 2,4-dihydroxybenzaldehyde were increased while for mAOX3 and mAOX2 the *k*_*cat*_ remained constant. We also observed that the effect of hydroxyl groups on the activity and affinity of mAOX1 was opposite to that observed for the other enzymes, revealing that the composition of the active site influences the substrate specificity and selectivity in respect to the orientation of the hydroxyl group in the active site.

The kinetic parameters of mAOX1, mAOX3, and mAOX2 mainly remained comparable with methoxybenzaldehydes as substrate independent on whether the methoxy-group is present in the ortho-, meta- or para- position. As mentioned above, mAOX4 was inactive with any methoxybenzaldehyde or ethylvanillin, showing a more narrow substrate binding site being unable to accommodate a methoxy group or ethoxy group at position 3. Surprisingly, vanillin was used as a substrate by mAOX4, however, with a slow substrate turnover of 15.9 min^-1^. This shows that the -OH group at position 4 of vanillin is important for substrate binding. For mAOX2, in contrast, the activity with methoxybenzaldehyde was almost 3-fold decreased when the methoxy group was present at the ortho position. It can also be concluded that hydroxy-, nitro-, or methoxy- groups at the para position did not considerably affect the activity of mAOX2 with any of the substrates. However, mAOX2 exhibited a ~6 times higher *k*_*cat*_ and ~20 times lower *K*_*M*_ with salicylaldehyde as compared to 2-methoxybenzaldehyde, showing that the ligand at the para position indeed affects the substrate turnover depending on the size of the ligand.

Previously, partially purified guinea pig liver aldehyde oxidase was analyzed for its activity towards structurally related benzaldehydes in a report by Panoutsopoulos and Beedham [27]. In this report the lowest *K*_*M*_ values were obtained with 2-methoxy-, 3-hydroxy-, and 4-hydroxybenzaldehydes. Here, we also observed a relatively low *K*_*M*_ value (less than 2μM) with 4-hydroxybenzaldehyde for mAOX3, mAOX4, and mAOX2. Similarly, the *K*_*M*_ value of mAOX1 and mAOX2 for 2-methoxybenzaldehyde was the lowest value among all benzaldehyde derivative substrates tested. As for mAOX3, substrate affinity was highest for all methoxybenzaldehydes even though the turnover numbers were similar to those of mAOX1. This shows that the substrate specificity of the AOX isoenzymes might be comparable among different species, however, considering that in the report by Panoutsopoulos and Beedham [27] a mixture of the three AOX1, AOX4 and AOX2 isoenzymes present in the liver extract were analyzed.

### N-Heterocyclic compounds

Human AOX (hAOX1) has been described to be involved in phase I drug metabolism [11] and its ability to oxidize drug molecules is associated with the ability to oxidize compounds containing N-heterocyclic ring structure. Based on this activity of the human enzyme we analyzed the N-heterocyclic compounds phthalazine, phenanthridine, and purine derivatives as substrates for the mAOX isoenzymes.

**Fig 2:**
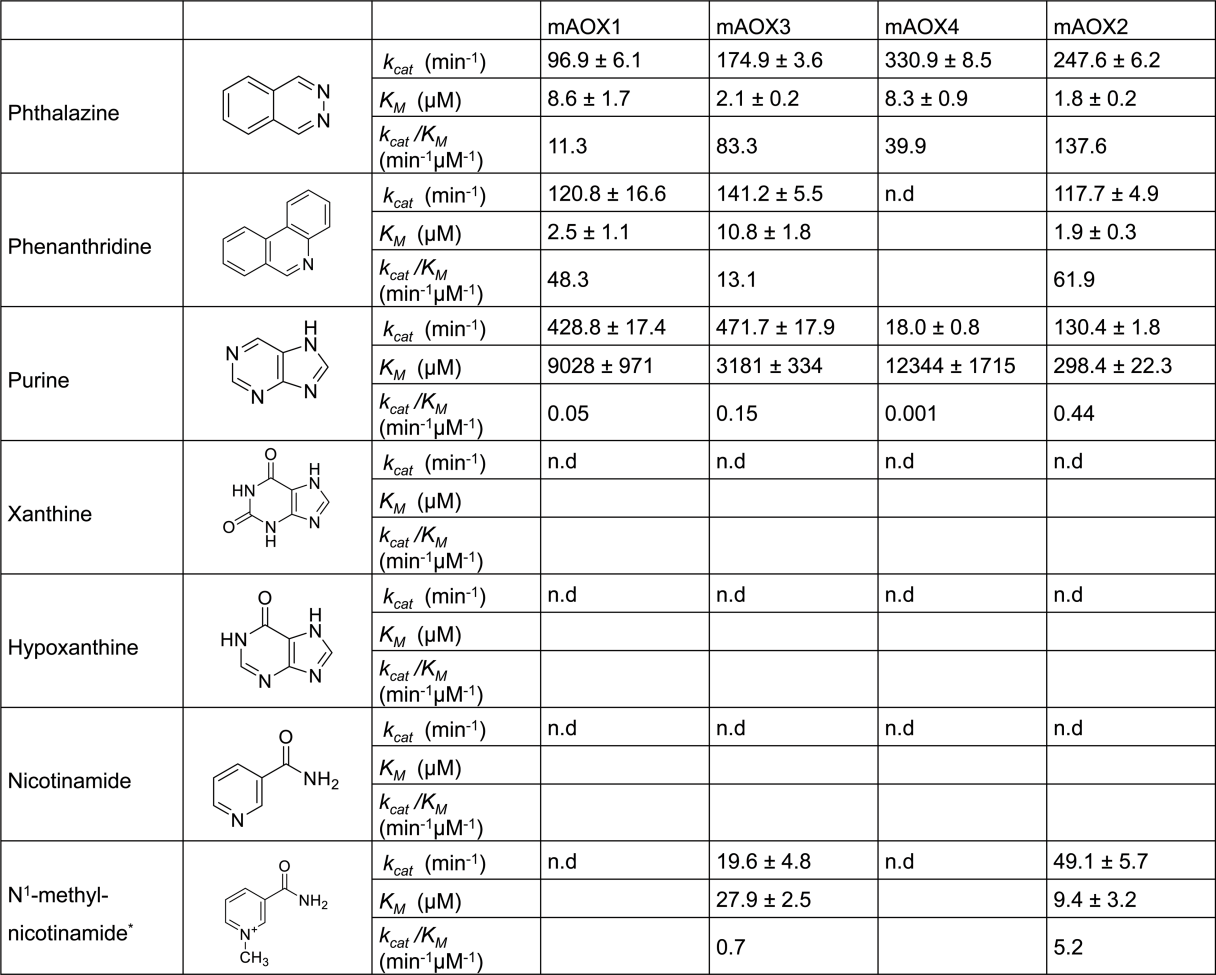
Steady-state kinetic parameters for mAOX1-4 with N-heterocyclic compounds as substrates. Apparent steady-state kinetic parameters were recorded in 50 mM Tris-HCl, 200 mM NaCl, and 1 mM EDTA (pH 8.0) in the presence of 100 μM DCPIP as electron acceptor. The substrate concentrations were varied around 0.5 and 10 times the K_M_. The chemical structure of each substrate is shown in the Fig. The values were corrected to a molybdenum saturation of 100% for each mAOX variant for a better comparability. Kinetic Data are mean values from three independent measurements (±S.D.). n.d. = no activity detectable.

The results in Fig 2 show that the oxidation of phthalazine by mAOX3, mAOX4, and mAOX2 was more efficient as compared to mAOX1 both in respect to *k*_*cat*_ and *K*_*M*_, with mAOX4 showing the highest k_*cat*_ value among the four AOX isoenzymes. Generally, the obtained *K*_*M*_ values for mAOX1 using phthalazine were in the same order of magnitude as compared to previously reported *K*_*M*_ values using human liver cytosol fractions [28], heterologously expressed mAOX1 [26] or mAOX3 [29], while other studies obtained lower *K*_*M*_ values also using human liver cytosol extracts [30]. Further, the *k*_*cat*_ for the phthalazine oxidizing activity of mAOX3 purified from mouse livers was similar to the value obtained in this study, showing the comparability between the activity of the recombinant enzyme and the one from a native source [25].

With phenanthridine as substrate, the activities of mAOX1, mAOX3, and mAOX2 were comparable to each other with *k*_*cat*_ values of 120.8 min^-1^, 141.2 min^-1^, and 117.7 min^-1^, respectively. Surprisingly, phenanthridine was not used as a substrate for mAOX4 in our study, while a study by Terao et al. [7] reported on the phenanthridine oxidation activity of mAOX4. In that study, HEK293 cell lines were transfected with the plasmids containing *mAox4* cDNA and protein extracts were used to measure AOX activity. Therefore, it is possible that phenanthridine oxidizing activity may not directly result from the mAOX4 enzyme but from cross contaminations of mAOX1 from the HEK cells.

AOX enzymes were reported previously to be able to oxidize purines as substrates, but not xanthine and hypoxanthine, which are readily oxidized by xanthine oxidase [31]. As expected, none of the mAOX isoenzymes showed activity with xanthine and hypoxanthine as substrates, while purine was a substrate for all four enzymes (Fig 2). In the literature studies with purine as substrate for AOX are limited, but it was reported before that rabbit liver aldehyde oxidase can utilize purine with a high turnover number and a remarkably low *K*_*M*_ [31, 32]. In comparison, mAOX1 and mAOX3, which are mainly present in the liver showed high *k*_*cat*_ values (428.8 and 471.7 min^-1^, respectively) and *K*_*M*_ values in the millimolar range. For mAOX4 also a high *K*_*M*_ value of 12344 μM was obtained, however, the enzyme showed a low *k*_*cat*_ value of only ~4% in comparison to the one obtained for mAOX1 and mAOX3. Among the four mouse enzymes, the highest catalytic efficiency was observed for mAOX2, which resulted from the relatively low level of *K*_*M*_ value (298.4 μM).

N^1^-methylnicotinamide, a primary metabolite of nicotinamide degradation, is metabolized to N^1^-methyl-2-pyridone-5-carboxamide (2-PY) or N^1^-methyl-4-pyridone-5-carboxamide (4-PY) by AOX [33]. This substrate was compared in a previous study by us and is only listed here for completenes [21]. To summarize the previous results, we observed a notable difference in the activity of N^1^-methylnicotinamide, as it was only reactive with mAOX3 and mAOX2 but not with mAOX1 and mAOX4. Here, we tested in addition whether nicotinamide is a substrate for mAOX isoenzymes. The results in Fig 2 show, however, that nicotinamide is not used as a substrate by any of the four mAOX isoenzymes.

In summary, the results show that mAOX4 is unable to react with phenanthridine, N^1^-methylnicotinamide and has only a poor activity with purine, all of which are good substrates for other mouse AOX enzymes. Thus, a physiological role of mAOX4 in the oxidation of aromatic heterocyclic compounds can be excluded. Recently, it has been reported in a study on the metabolomic profiling of the Harderian gland that tryptophan is an endogenous substrate for mAOX4 [6]. The authors reported that the indole side chain of tryptophan is the target for AOX4 activity and the resulted mono-hydroxylated products are different than 5-OH-tryptophan, the product of tryptophan-hydroxylases. In contrast to this report, all attempts to detect activity with tryptophan for mAOX4 failed in our hands. Either the reaction of mAOX4 with tryptophan is too slow to be detected in our assay, M, which then can serve as a substrate for mAOX4. Further investigations are necessary to clarify this.

### Alkyl (aliphatic) aldehydes

**Fig 3:**
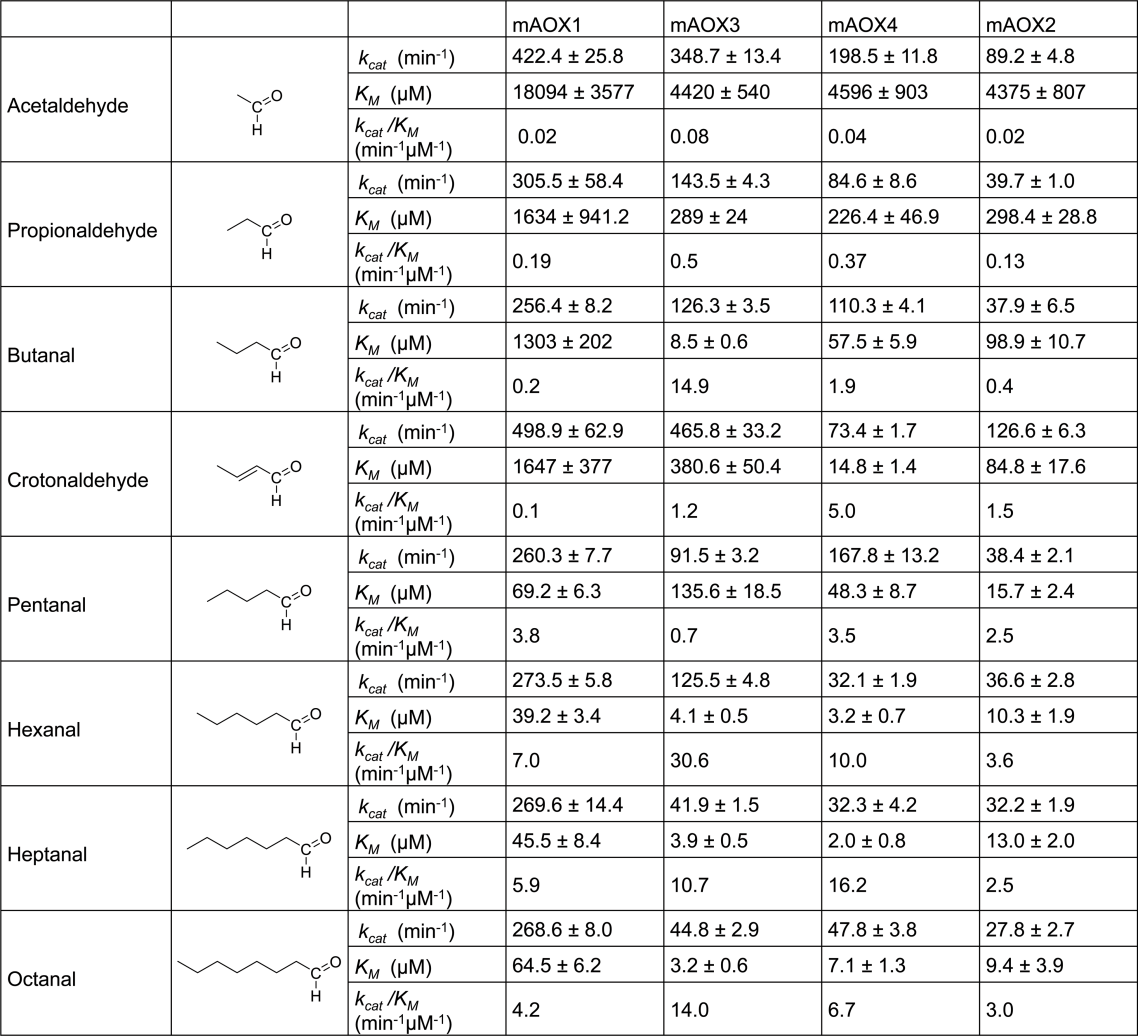
Steady-state kinetic parameters for mAOX1-4 with aliphatic aldehydes as substrates. Apparent steady-state kinetic parameters were recorded in 50 mM Tris-HCl, 200 mM NaCl, and 1 mM EDTA (pH 8.0) in the presence of 100 μM DCPIP as electron acceptor. The substrate concentrations were varied around 0.5 and 10 times the K_M_. The chemical structure of each substrate is shown in the Fig. The values were corrected to a molybdenum saturation of 100% for each mAOX variant for a better comparability. Kinetic Data are mean values from three independent measurements (±S.D.). n.d. = no activity detectable.

The catalytic activities of mAOX isoenzymes were further examined with aldehydes containing aliphatic carbon chains as substrates. Generally, higher *K*_*M*_ values were obtained for more hydrophilic substrates such as acetaldehyde and propionaldehyde (Fig 3). This is also in agreement with the high *K*_*M*_ values obtained with purine, which is also hydrophilic. Thus, the substrate-binding funnel seems to have a more hydrophobic nature in all mAOX enzymes.

Acetaldehyde is a toxic intermediate in the metabolism of methanol. In our assays, however, the *K*_*M*_ values for acetaldehyde were relatively high for all mAOX enzymes with values around 4.4–4.6 mM for mAOX3, mAOX4, and mAOX2 and a value of 18 mM for mAOX1. In consistency, a study by Villa et al. [5] investigated the activities of mAOX1 and mAOX3 purified from mouse livers and reported that both enzymes are relatively inefficient metabolizers of acetaldehyde. No significant differences in the acetaldehyde concentration in the liver of mAOX1/mAOX3 deficient mice was observed after direct injection of ethanol [5]. This implies that mAOX enzymes are likely not directly involved in the metabolism of acetaldehyde and that this reaction is rather catalyzed by aldehyde dehydrogenases (ALDH2 and ALDH1A1), oxidizing acetaldehyde to acetate [34].

The alkyl aldehydes tested in this study showed the highest turnover numbers for mAOX1. The length of the carbon chain thereby had no significant effect on the *k*_*cat*_ values for mAOX1 with values around 250 min^-1^. However, also the *K*_*M*_ values for mAOX1 were very high for the alkyl substrates. Overall, mAOX2 showed the lowest catalytic efficiency for each of the substrates with the exception of crotonaldehyde. In comparison to mAOX4, the activities with hexanal, heptanal and octanal were similar, giving equivalent turnover numbers and *K*_*M*_ values. Also mAOX3 showed very high *K*_*M*_ values for all substrates. Hexanal was nevertheless 2-fold better substrate than hexanal and heptanal for mAOX3.

The *K*_*M*_ values generally decreased for all enzymes with a longer carbon chain of the substrate. Especially *K*_*M*_ values in the low micromolar range were obtained with the aldehydes containing 5 or longer carbon chain (with the exception of mAOX3 for pentanal), indicating that the substrate affinity with the medium-chain aldehydes was increased. The increase in substrate affinity, however, resulted in a drop of the turnover number especially for mAOX3, mAOX4, and mAOX2. A similar observation with a decrease in the activity and the increase in substrate affinity with increasing the alkyl chain length of the substrate has been also reported for aldehyde dehydrogenase enzymes [35]. In cytosolic ALDH1 the *K*_*M*_ value decreased from 180 μM to 0.012 μM with moving from acetaldehyde to octanal as substrate. For mitochondrial ALDH2, a 7-fold decrease in the *K*_*M*_ for acetaldehyde (0.2 μM) to the *K*_*M*_ for octanal (0.028 μM) was reported [35]. For both enzymes, the *k*_*cat*_ for octanal was decreased 3.2 to 1.3-fold when compared to that of acetaldehyde. Thus, both mAOX and ALDH classes of enzymes share conserved features in the binding and conversion of hydrophobic aldehydes, however, the physiological relevance of mAOX enzymes in converting alkyl aldehydes remains to be determined. Overall, no large differences were observed in the conversion of alkyl aldehydes between the four mAOX enzymes.

### Cinnamaldehyde-related compounds

**Fig 4:**
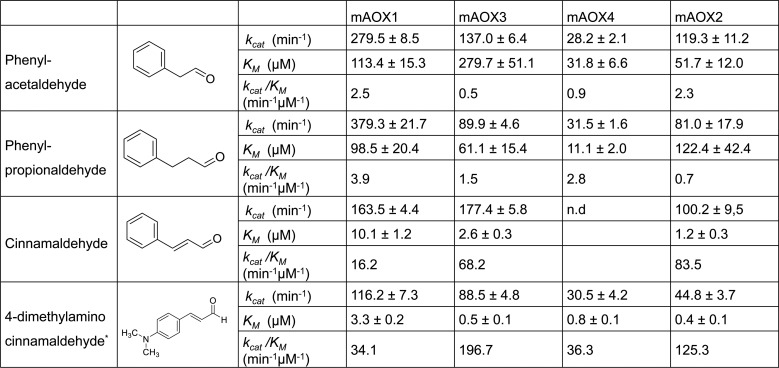
Steady-state kinetic parameters for mAOX1-4 with cinnamaldehyde-related compounds as substrates aromatic aldehydes as substrates. Apparent steady-state kinetic parameters were recorded in 50 mM Tris-HCl, 200 mM NaCl, and 1 mM EDTA (pH 8.0) in the presence of 100 μM DCPIP as electron acceptor. The substrate concentrations were varied around 0.5 and 10 times the K_M_. The chemical structure of each substrate is shown in the Fig. The values were corrected to a molybdenum saturation of 100% for each mAOX variant for a better comparability. Kinetic Data are mean values from three independent measurements (±S.D.). n.d. = no activity detectable.

Kinetic parameters of the four mAOX isoenzymes were also compared to the structurally related compounds phenylacetaldehyde, phenylpropionaldehyde, cinnamaldehyde, and 4-dimethylaminocinnamaldehyde (DMAC). The kinetic data for DMAC were reported previously and are listed here for a better direct comparison [21]. As shown in Fig 4, DMAC was the most efficient (*k*_*cat*_/*K*_*M*_) substrate for all mAOX isoenzymes. Cinnamaldehyde was also a good substrate for mAOX1, mAOX3, and mAOX2, but not for mAOX4, for which no substrate conversion could be detected. Overall, the turn-over numbers for all cinnamaldehyde-related substrates listed here were very low for mAOX4 with *k*_*cat*_ values around 30 min^-1^. In general, mAOX1 and mAOX3 showed the highest activities with the cinnamaldehyde-related compounds.

Phenylacetaldehyde is a naturally occurring metabolite during the L-phenylalanine metabolism, via the oxidation of 2-phenylethylamine by monoamine oxidase. The oxidation of phenylacetaldehyde to phenylacetate was shown to be catalyzed by ALDH [35]. The reaction of phenylacetaldehyde with ALDH1 and ALDH2 has been reported with *K*_*M*_ values of 5.5 and 0.03 μM, respectively [35]. Panoutsopoulos et. al. [36] reported that also AOX and XO catalyze the oxidation of phenylacetaldehyde and phenylacetaldehyde was shown previously to be a good substrate for rat liver AOX with a *K*_*M*_ value of 53 μM and a *V*_*max*_ value of 0.44 μmol/min/mg. Our data are in agreement with the literature showing that phenalacetaldehyde can be used as a substrate by all four mAOX enzymes. However, the *k*_*cat*_ values of 3000 min^-1^ and 1800 min^-1^ for ALDH1 and ALDH2, respectively, are much higher and show that both enzymes are the major metabolizer of phenylacetaldehyde in the cell [35]. It can be concluded that AOX might have a specific role in the conversion of phenylacetaldehyde under specific cellular conditions, which should be investigated further in the future for its physiological relevance.

By a more detailed comparison of the kinetic parameters of the cinnamonaldehyde-related compound and the alkyl-aldehydes, it becomes obvious that the presence of a dimethylamino group (i.e., DMAC), a double bond between the C2 and C3 of the side chain (i.e., cinnamaldehyde) or the length of the side chain (i.e., phenyl-acetaldehyde, -propionaldehyde) can have different effects on the kinetic parameters of mAOX isoenzymes. In particular, mAOX3 and mAOX4 showed lower *K*_*M*_ values for phenylpropionaldehyde in comparison to phenylacetaldehyde, while the effect was opposite for mAOX2, which showed ~2 fold incresed *K*_*M*_ with phenylpropionaldehyde vs. phenylacetaldehyde. The *k*_*cat*_ values thereby were comparable. A significant difference in activity became obvious by comparison of mAOX1 and mAOX3. With phenylpropionaldehyde a *k*_*cat*_ value of 379.3 min^-1^ was obtained for mAOX1, while with cinnamaldehyde the *k*_*cat*_ value was significantly lower with 163.5 min^-1^. For mAOX3 the numbers were reverted with a *k*_*cat*_ of 90 for phenylpropionaldehyde and a *k*_*cat*_ of 177 for cinnamaldehyde. Overall, mAOX4 displayed the lowest activity with the cinnamaldehyde-related compounds, while no major difference in the selectivity of these aldehydes was observed between mAOX1, mAOX3 or mAOX2.

### Site-directed mutagenesis of amino acids that determine the selectivity of mAOX4

Overall, the results of the substrate specificities of the four mAOX isoforms show that generally mAOX1 is the most active enzyme for most substrates and no real substrate specificity pattern can be assigned for mAOX, mAOX3 or mAOX2. mAOX4, however, is generally the least active enzyme with most substrates and shows also the highest selectivity, since some substrates are not converted by mAOX4, especially more hydrophilic ones. To investigate the factors that determine the substrate selectivity of mAOX4 in more detail, site directed mutagensis of mAOX4 was performed and selected amino acids in the substrate-binding funnel and at the active site of mAOX4 were exchanged to the ones present in the other mAOX isoforms.

**Fig 5:**
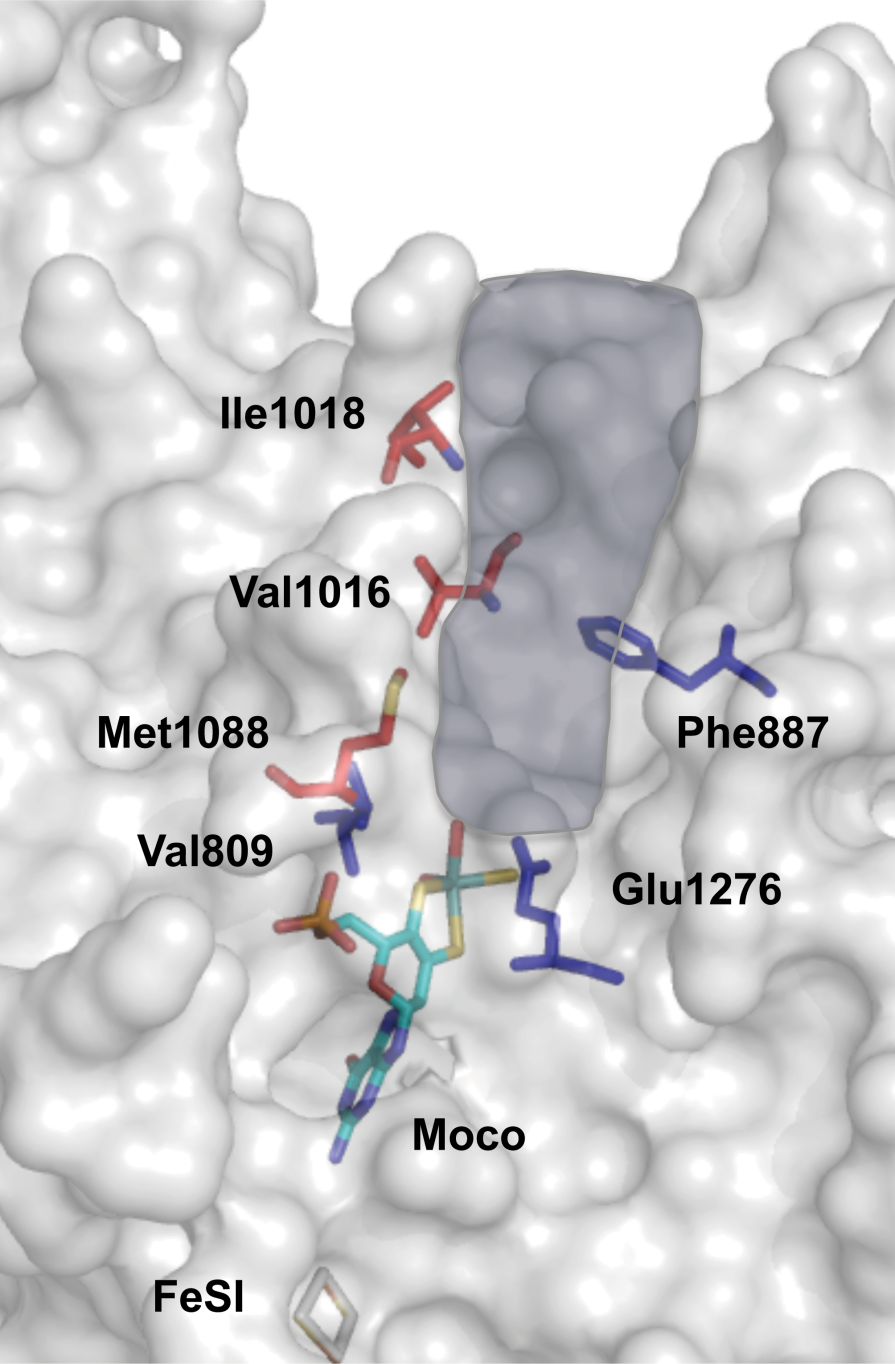
Active site and substrate-binding tunnel in mAOX4. Shown is a model of the structure of mAOX4 active site generated with Pymol using hAOX1 as template. Shown is the surface-representation of the substrate-binding site of mAOX4 with highlighted residues in the conserved region of the active site and in the non-conserved region which dictate substrate specificity. The substrate-funnel is highlighted in blue according to the study by Cerqueira et al. [20]. Residues in *red* indicate the amino acids whose nature only specific for mAOX4. Residues in *blue* are directly involved in substrate binding or in initiating the catalytic mechanism. The Moco and FeSI is shown in stick representation.

We selected the amino acids Val1016, Ile1018 and Met1088 as targets for amino acid exchanges by site-directed mutagenesis to identify their specific role in substrate-binding and conversion (Fig 5). The corresponding amino acids in the other three mAOX isoforms are listed in Table 1. In general, these residues are not conserved and were identified to be specific for mAOX4. Val1016 was exchanged to a leucine present in human AOX1, to a phenylalanine present in mAOX3, mAOX2 and bXDH enzymes, and to an isoleucine present in mAOX1. Ile1018 was exchanged to a lysine present in mAOX3 and to a serine present in human or mouse AOX1. Met1088 was exchanged to a threonine present in mAOX3 and a valine present in mouse and human AOX1, in mAOX2 and bXDH. Previously, the mAOX4-Val1016 corresponding residue in mAOX3 (Phe1014) and bXDH (Phe1009), were identified to be responsible for the correct alignment and orientation of substrate [17, 37]. In bXDH, the pyrimidine ring of urate is stacked in the middle between Phe1009 and Phe914 (bXDH numbering). Ile1018 is located at the entrance of active site funnel and marked as the enzyme specific amino acid residues in mAOX1 (Ser1015) and mAOX3 (Lys1016) [20]. While Met1088 is not directly exposed to the funnel or interacting with substrate or inhibitors, it is present in the interior of the funnel and identified as an important mAOX4 specific residue [20].

**Table 1:** Comparison of the active site amino acid residues of hAOX1, mAOX1, mouse AOX3, mAOX4, mAOX2, and bXDH which were shown to be specific to mAOX4 [20].

mAOX1	mAOX3	mAOX4	mAOX2	hAOX1	bXDH
Ile 1013	Phe 1014	Val 1016	Phe 1024	Leu 1018	Phe 1009
Ser 1015	Lys 1016	Ile 1018	Ala 1026	Ser 1020	Val 1011
Val 1085	Thr 1086	Met 1088	Val 1096	Val 1090	Val 1081

**Fig 6:**
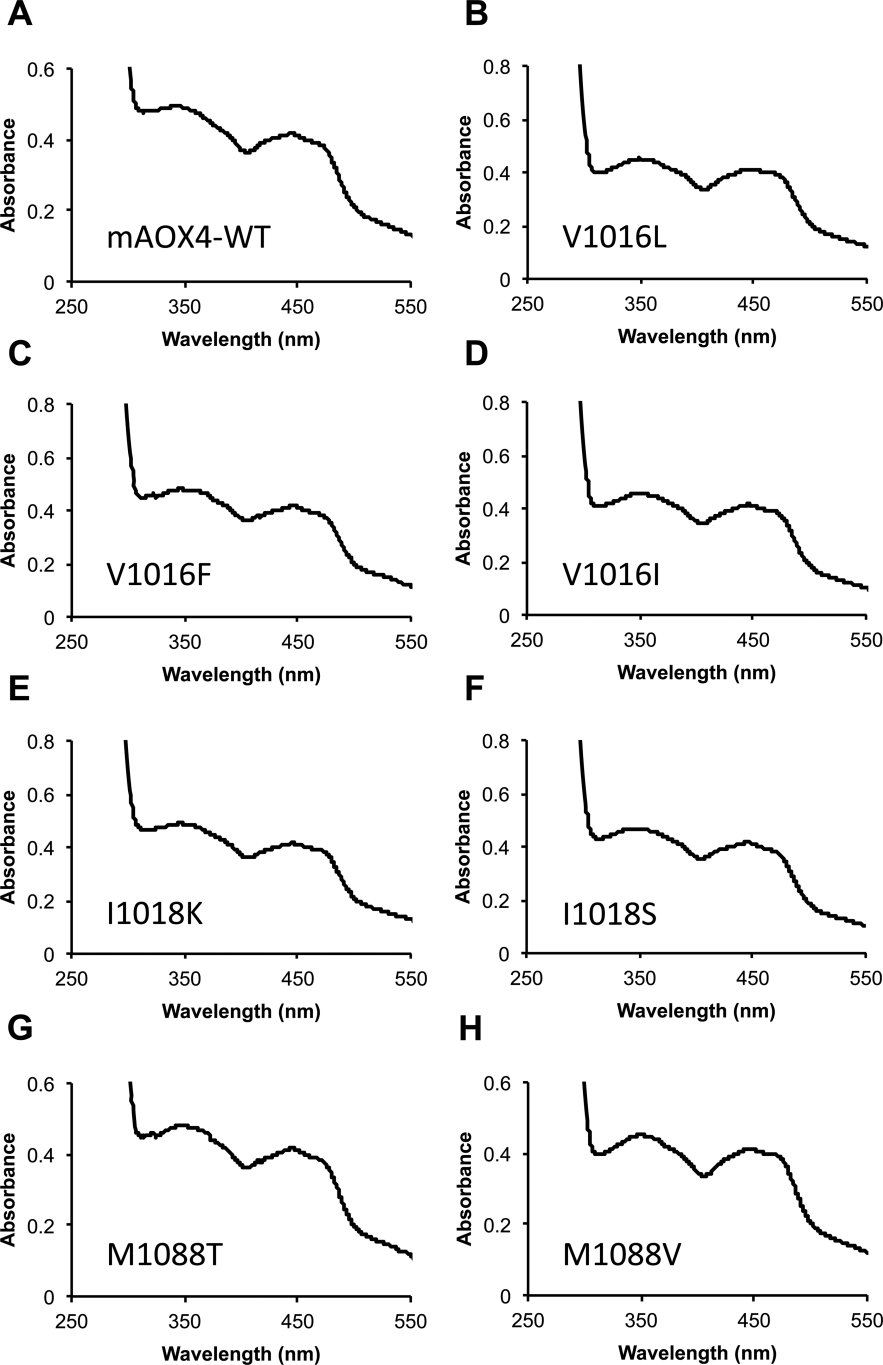
UV-Vis spectra of mAOX4 variants in comparison to mAOX4-wild-type. The Fig illustrates UV-Vis spectra of 10 μM mAOX4 wild-type and variants (as indicated) in the oxidized state recorded in 50 mM Tris, pH 8.0.

**Fig 7:**
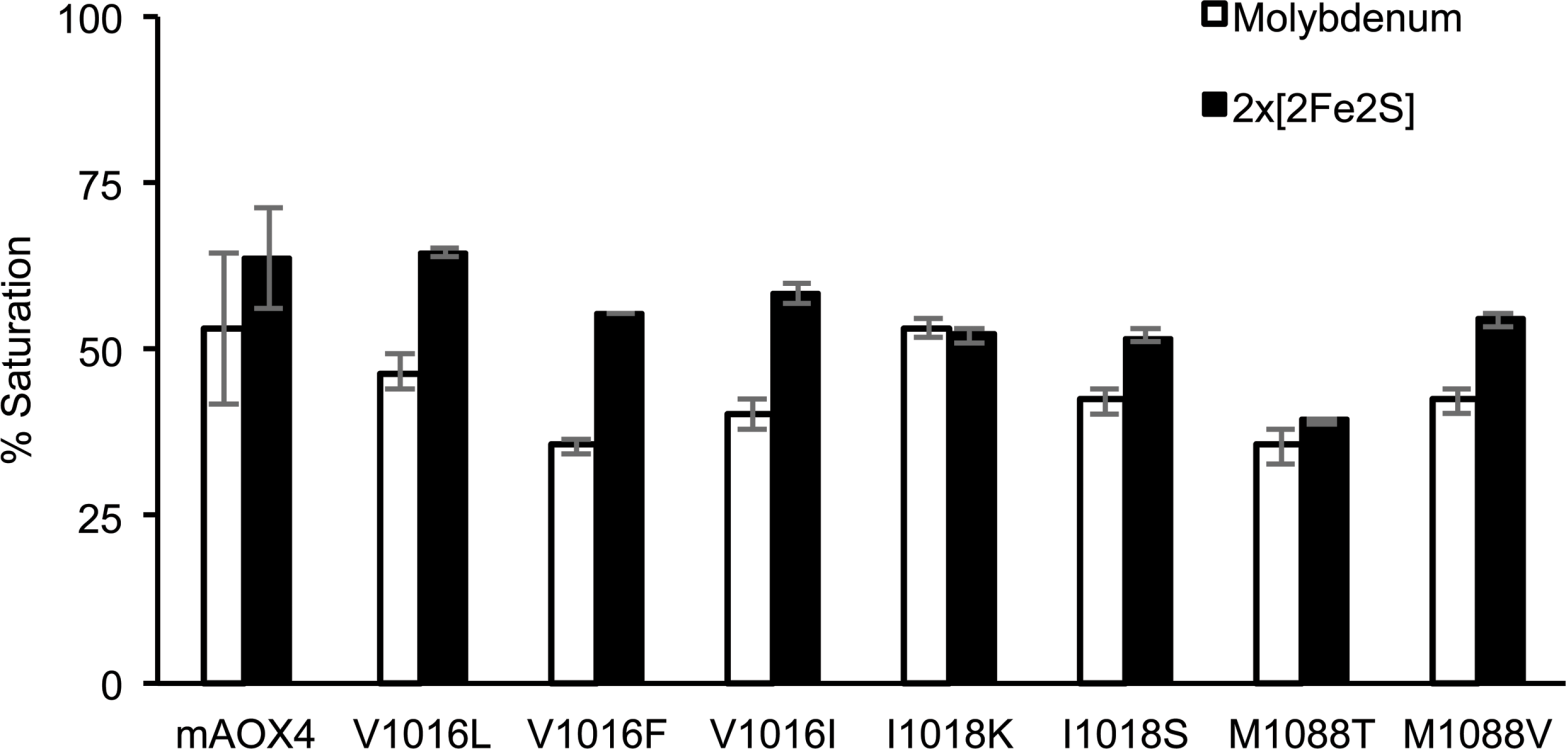
Molybdenum and iron saturation of purified mAOX4 variants. After purification, the molybdenum and iron content of the indicated enzymes were determined by inductively coupled plasma optical emission spectroscopy. The iron content corresponds to saturation with both, FeSI and FeSII clusters (corresponding to four molecules of Fe). The 100% values are set to a full saturation of the enzyme with of Moco and 2x[2Fe-2S] clusters.

Expression and purification of the mAOX4 mutants were similar to the those of the wild-type enzyme [21], the absorption spectra of the purified variants are shown in Fig 6. After purification, all variants were subjected to chemical sulfuration, to obtain proteins with a comparable saturation of the sulfido-group [21]. Saturation of the purified mAOX4 variants with Moco and FeS clusters was determined by measuring the molybdenum and iron contents of the proteins as described previously (Fig 7) [21]. In general, the saturation of the variants with molybdenum was comparable to the wild-type protein with the exception of variants V1016F and M1088T, for which a lower molybdenum saturation of around 35% was determined. The iron saturation of the variants was more comparable among each other with saturation levels around 50–64%. In general, this shows that the mAOX4 variants were purified with comparable levels of bound cofactors so that a direct comparison of the kinetic parameters are feasible. For better comparison, the kinetic parameters for the mAOX4 variants were determined with benzaldehyde, salicylaldehyde, phthalazine, and pentanal as substrates and corrected to a 100% molybdenum saturation for better comparability (Table 2).

**Table 2:** Steady state kinetic parameters mAOX4 wildtype and variants with different substrates. Steady-state kinetics were corrected to molybdenum saturation of 100%. Kinetic parameters were recorded in 50 mM Tris-HCl, 200 mM NaCl, and 1 mM EDTA (pH 8.0) in the presence of 100 μM DCPIP as electron acceptor. Substrate concentration were varied around 0.5 and 10 times the K_M_. Data are mean values from three independent measurements (±S.D.).

Enzyme		Benzaldehyde	Salicylaldehyde	Phthalazine	Pentanal
mAOX4-WT	*k*_*cat*_ (min^-1^)	91.5 ± 2.3	205.2 ± 6.7	330.9 ± 8.5	167.8 ± 13.2
	*K*_*M*_ (μM)	2.2 ± 0.2	13.2 ± 2.4	8.3 ± 0.9	48.3 ± 8.7
	*k*_*cat*_ */K*_*M*_ (min^-1^μM^-1^)	41.6	15.6	39.9	3.5
mAOX4-V1016L	*k*_*cat*_ (min^-1^)	28.7 ± 1.0	55.9 ± 2.3	13.0 ± 0.4	±
	*K*_*M*_ (μM)	5.4 ± 1.1	17.4 ± 2.9	27.6 ± 6.6	±
	*k*_*cat*_ */K*_*M*_ (min^-1^μM^-1^)	5.3	3.2	0.5	
mAOX4-V1016F	*k*_*cat*_ (min^-1^)	39.8 ± 0.5	23.6 ± 2.8	40.2 ± 2.1	66.5 ± 5.4
	*K*_*M*_ (μM)	1092 ± 47	3886 ± 887	1264 ± 188	5566 ± 918
	*k*_*cat*_ */K*_*M*_ (min^-1^μM^-1^)	0.04	0.01	0.03	0.01
mAOX4-V1016I	*k*_*cat*_ (min^-1^)	23.4 ± 0.5	34.8 ± 1.0	16.5 ± 0.2	27.1 ± 0.4
	*K*_*M*_ (μM)	5.1 ± 0.5	44.7 ± 6.1	10.6 ± 0.8	29.3 ± 2.5
	*k*_*cat*_ */K*_*M*_ (min^-1^μM^-1^)	4.6	0.8	1.6	0.9
mAOX4-I1018K	*k*_*cat*_ (min^-1^)	68.6 ± 2.5	113.9 ± 4.5	114.4 ± 1.5	76.8 ± 1.7
	*K*_*M*_ (μM)	2.1 ± 0.3	8.1 ± 1.5	7.7 ± 0.4	13.4 ± 1.1
	*k*_*cat*_ */K*_*M*_ (min^-1^μM^-1^)	32.7	14.1	14.9	5.7
mAOX4-I1018S	*k*_*cat*_ (min^-1^)	75.0 ± 2.3	307.4 ± 19.3	317.6 ± 6.2	158.7 ±7.3
	*K*_*M*_ (μM)	1.1 ± 0.2	9.2 ± 3.2	5.9 ± 0.6	13.4 ± 2.2
	*k*_*cat*_ */K*_*M*_ (min^-1^μM^-1^)	68.2	33.4	53.8	11.8
mAOX4-M1088T	*k*_*cat*_ (min^-1^)	319.4 ± 27.2	675.2 ± 17.9	618.1 ± 20.8	479.0 ± 75.2
	*K*_*M*_ (μM)	6.4 ± 2.2	23.8 ± 1.8	9.4 ± 1.2	57.0 ± 16.9
	*k*_*cat*_ */K*_*M*_ (min^-1^μM^-1^)	49.9	28.4	65.8	8.4
mAOX4-M1088V	*k*_*cat*_ (min^-1^)	35.3 ± 0.7	68.5 ± 2.1	62.3 ± 0.9	55.2 ± 1.3
	*K*_*M*_ (μM)	0.8 ± 0.1	13.6 ± 1.7	3.3 ± 0.2	28.4 ± 2.2
	*k*_*cat*_ */K*_*M*_ (min^-1^μM^-1^)	44.1	5.0	18.9	1.9

The results obtained for the mAOX4-Val1016 variant reveal that major changes on the kinetic constants were obtained. In general, a 80% decrease in the activity was observed for all three V1016L, V1016F and V1016I variants in comparison to mAOX4 wild-type. For the variants V1016L and V1016I, only minor changes in *K*_*M*_ were observed. However, by introducing a phenylalanine at position 1016, which is present in mAOX3, the *K*_*M*_ largely increased by two orders of magnitude, while the *k*_*cat*_ remained in a comparable range to the V1016L and 1016I variants (3–5 fold lower as the wild-type). Given the fact that Val1016 is predicted to be involved in substrate stabilization, the presence of highly hydrophobic and bulky residue like phenylalanine might cause perturbations at the active site especially for mAOX4, which was shown to have a higher substrate selectivity. Additionally, the aromatic group of phenylalanine might cause an unnatural stacking of the aromatic group of the substrates in mAOX4. In contrast, a previous report on mAOX3 showed that substituting the corresponding amino acid Phe1014 in mAOX3 by an isoleucine or valine resulted in a decrease in *k*_*cat*_ and *K*_*M*_, showing that the active site of mAOX3 was optimized to accommodate a bulky hydrophobic amino acid [29].

Exchange of Ile1018 to a lysine resulted in a 2-fold decrease in *k*_*cat*_, while *K*_*M*_ remained unaffected with benzaldehyde, salicylaldehyde, and phthalazine as substrates. The *K*_*M*_ with pentanal, however, increased 3-fold with a simultaneous increase of *k*_*cat*_. In the I1018S variant, *K*_*M*_ was decreased 1.5-3-fold, while the *k*_*cat*_ values overall mainly remained unaffected with all substrates. These changes in the catalytic constants can be explained by the location of Ile1018 at the protein surface being exposed to the substrate funnel. It is likely that when it is replaced by a lysine, the positive charge of the α-amino group of the lysine side chain alters the substrates access to the cavity. Serine is rather slightly polar and a small amino acid and as expected the effect on activity was less pronounced than the lysine replacement.

A major effect on the kinetic constants was obtained for the M1088T variant. In this variant the *k*_*cat*_ was significantly increased 3-fold in comparison to the wild-type enzyme. The *K*_*M*_ values remained mainly unaffected for phthalazine and pentanal as substrates, while the *K*_*M*_ values for benzaldehyde and salicylaldehyde were 2-3-fold increased. In the M1088V variant in contrast, the *k*_*cat*_ values were reduced to half of the activities of the wild-type enzyme, while the *K*_*M*_ values mainly remained unchanged or were also 50% reduced.

Generally, M1088T mutant was the only variant for that high level of activity increase was observed compared to the wild-type. All the other variants exhibited 2 to 3-fold decrease in substrate conversion. All the variants were also assayed for activity with substrates for which no activity was obtained for mAOX4, such as 2-methoxybenzaldehyde, vanillin, phenanthridine, N^1^-methylnicotinamide, and cinnamaldehyde. In these assays, the catalytic activity of the M1088T variant was solely increased with vanillin, resulting in a *k*_*cat*_ value of 51.9 min^-1^, while also this variant was inactive with the other substrates. Thus, the M1088T exchange largely affected the activity of mAOX4. Threonine was identified to be present in mAOX3 at the corresponding position, the AOX variant that in general showed higher activities as compared to mAOX4 for most substrates tested in this report. This implies that mAOX4 has evolved as an isoenzyme with low activity, which might be beneficial for the role of the enzyme in the Harderian gland.

## Conclusions

The results in this manuscript show that in general, the substrate specificities of mAOX1, mAOX3 and mAOX2 are overlapping and no real substrate specificity pattern can be assigned for each isoform. The only exception is mAOX4, which shows the lowest activity with all substrates and the highest selectivity. mAOX4 can not react with more hydrophilic aromatic substrates and some N-heterocyclic compounds, in consistency with the predicted hydrophobic substrate-binding funnel [20]. The role of mAOX4 is specific to the Harderian gland, however, the enzyme has not evolved for high substrate turnover in this tissue, since one selected amino acid exchange in an amino acid specific for mAOX4 was leading to an increase in activity, while the substrate selectivity was unaltered in this variant. From the overlapping substrate specificities of mAOX1, mAOX3 and mAOX2, mAOX1 is the most effective enzyme with almost all substrates tested in this study. Both mAOX1 and mAOX3 are mainly expressed in the liver and have evolved overlapping substrate specificities with high substrate turnover rates. This role seems to be beneficial in the liver, where most substrate degradation pathways occur. The role of mAOX2 in rodents still needs to be elucidated in more detail in the future. Expression of mAOX2 is highly restricted to the Bowman's gland in the nasal cavity, but no specific substrate for mAOX2 could be identified within this study, and the substrate specificities are overlapping with those of mAOX1 and mAOX3. In future studies, crystal structures for all four mAOX isoforms need to be determined to reveal differences of the enzymes at the structural level in more detail. Additionally, metabolomic studies with knock out mice in the specific isoforms might give more hints on the specific role of each isoform in the cellular metabolism.

Overall, for drug clearance prediction studies in humans, we propose to perform the studies directly on the human system. As predicted before, due to the existence of four different AOX enzymes in mice and rats and a single AOX enzyme in humans, the use of rodents as pre-clinical models for pharmacokinetic, pharmacodynamic and toxicological studies involving drugs which are potential human AOX1 substrates are generally not suitable model systems for these particular studies [21, 38]. Even though the four mouse isoforms are more similar among each other than previously predicted by computational studies, significant differences to the human enzyme exist, which should be considered carefully.

## Materials and methods

### Expression and purification of mAOX enzymes

*E*. *coli* TP1000 *(∆mobAB*) cells [39] were used for the expression of mAOX enzymes from plasmids pMAOX1co, pMMA1, pMAOX4, and pMAOX2co, which are described in more detail in [21]. *E*. *coli* cultures were grown in 2 Liter flasks containing 1 Liter of LB-peptone supplemented with 150 μg/mL ampicillin, 1 mM Na_2_MoO_4_ and 20 mM isopropyl β-D-thiogalactoside (IPTG) at 30°C and 130 rpm. Expression was generally performed in 12 Liter volumes in total. Cells were harvested after 24 hours of growth by centrifugation at 5000 x g for 5 minutes, and the pellet was resuspended in 50 mM sodium phosphate buffer pH 8.0, containing 300 mM NaCl. Cell lysis was achieved by two consecutive passages through a cell disruptor at 12°C and 1.35 kbar (TS Benchtop Series Constant Systems, Northampton, UK). 1 mg/ml DNase I was added to cell lysate after the first cycle. The cell debris was removed by centrifugation at 18000 g for 1 h at 4°C, and the supernatants were assayed to protein purification.

For protein purification, the supernatants after cell lysis were transferred into Ni-NTA (Macherey and Nagel, Düren, Germany) columns and passed through 0.3 ml of resin per liter of culture two times. The matrix was washed first with 20 column volumes of 50 mM sodium phosphate and 300 mM NaCl (pH 8.0) containing 10 mM imidazole, then the same buffer containing 20 mM imidazole was applied. Proteins were eluted from the matrix with 50 mM sodium phosphate and 300 mM NaCl (pH 8.0) containing 250 mM imidazole. For further purification, the buffer of partially purified enzymes was changed to 50 mM Tris-HCl, 200 mM NaCl and 1 mM EDTA (pH 8.0) by using PD-10 columns (GE Healthcare, Chalfont St. Giles, Buckinghamshire, UK). Final purification was obtained by the use of size exclusion chromatography on Superdex 200 column (GE Healthcare) equilibrated in 50 mM Tris-HCl, 200 mM NaCl and 1 mM EDTA (pH 8.0). Elution profiles were recorded by monitoring of the absorbance at 280, 450 and 550 nm and the fractions containing AOX were analyzed by SDS-polyacrylamide gel electrophoresis (PAGE). The ones containing dimeric AOX with a high purity were combined. Purified enzymes were subjected to an *in vitro* chemical sulfuration step purification using a protocol described previously [21] directly after the size exclusion chromatography. If necessary, proteins were concentrated by ultrafiltration (Amicon 50-kDa MW cut-off; Milipore Corporation, Billerica, MA).

### Enzyme kinetics assays

Enzyme assays were carried out in 10 mm cuvettes in a buffer containing 50 mM Tris-HCl (pH 8.0) and 1 mM EDTA with a total volume of 800 μl at 37°C. For all measurement, it was assured that the substrates were used in a concentration range between 0.5 and 10 times of the calculated *K*_*M*_ value. 2,6 dichlorophenolindophenol (DCPIP) was chosen as the electron acceptor under saturating conditions at a concentration of 100 μM. DCPIP does not inhibit the mouse AOX enzymes. The total enzyme concentration varied between 0.05 and 0.2 μM, depending on the substrate and AOX enzyme used. The cuvettes containing substrate and DCPIP were incubated at 37°C prior to measurement using Grant Bio DB-10C dry block thermostat and the enzyme was added last to initiate the reaction. Enzymatic activity resulting from the reduction of DCPIP was followed at 600 nm for 1 minute with a computer-assisted spectrophotometer (Shimadzu UV-2600). The reaction was maintained at 37°C with a temperature-controlled cell holder (Shimadzu TCC-240A) coupled to the spectrophotometer. Absorbance change at the blank cuvette, which contained all the components except enzyme, was also measured to subtract the reduction of DCPIP that occurred by agents other than aldehyde oxidase. An extinction coefficient of 16100 M^-1^cm^-1^ was used for DCPIP to convert absorbance changes to the turnover number (*k*_*cat*_). Kinetic parameters were calculated by nonlinear fitting of the Michaelis-Menten equation. All fitting analyses were performed with the Origin software version 8.0 (OriginLab, Northampton, MA).
